# Deciphering the genomic and functional basis of virulence and immune modulation in *Pantoea stewartii*

**DOI:** 10.3389/fmicb.2026.1969847

**Published:** 2026-10-05

**Authors:** Jianying Liu, Na Peng, Xiaoxia Zhang, Hai-Lei Wei

**Affiliations:** Key Laboratory of Microbial Resources Collection and Preservation, State Key Laboratory of Efficient Utilization of Arable Land in China, Ministry of Agriculture and Rural Affairs, Institute of Agricultural Resources and Regional Planning, Chinese Academy of Agricultural Sciences (CAAS), Beijing, China

**Keywords:** flagellin polymorphism, MAMP triggered immunity, motility, *Pantoea stewartii*, type III secretion system

## Abstract

*Pantoea stewartii* subsp. *indologenes* is an emerging cereal phytopathogen with unclear virulence mechanisms. We sequenced a rice seed-borne strain, HR3-48, and classified it as *P. stewartii* subsp. *indologenes* (*Pnsi*) through phylogeny of 92 housekeeping genes, ANI (> 99%), and dDDH (> 96%) comparisons. HR3-48 harbors a conserved plant-pathogenic PSI-1 type III secretion system (T3SS) and a single flagellar locus (*flag-1*) with multiple *fliC* genes. The tandem *fliC456* cassette is functionally redundant for motility and flagellum assembly but contributes significantly to eliciting plant immune responses in *Nicotiana benthamiana*. The T3SS structural gene *hrcQ–U* determines hypersensitive response (HR) induction. HR3-48 causes necrosis in maize and rice, a phenotype primarily reliant on T3SS. The flagellin-derived epitope flg22^PanM^, a key polymorphism of L19M, triggered robust reactive oxygen species (ROS) production and inhibited *Pst* DC3000-induced HR in *N. benthamiana*, whereas flg22^PanE^ (L19E) showed weak or no activity in these early immune assays, indicating that the 19^th^ residue dictates the specificity of plant immune recognition. Both peptides suppress Arabidopsis seedling growth. Collectively, our data indicate that PSI-1 T3SS in *Pnsi* HR3-48 is a major virulence determinant in cereal hosts, whereas polymorphic flagellins modulate MTI (MAMP-triggered immunity) outputs and motility of HR3-48, with the 19^th^ residue of flg22 contributing to differential immune perception. It remains unclear how immune suppression is mediated by effectors.

## Introduction

*Pantoea stewartii* includes two subspecies. *P. stewartii* subsp. *stewartii* (*Pnss*), a host-specific xylem-dwelling phytopathogen that causes Stewart’s wilt in sweetcorn and maize (*Zea mays*) (Mergaert et al., 1993) and leads to worldwide economic losses in corn production (Jeger et al., 2018). The bacterium is transmitted by the corn flea beetle (*Chaetocnema pulicaria*) (Roper, 2011). While *P. stewartii* subsp. *indologenes* (*Pnsi*) has a broad host range, which causes millet leaf spot (Mergaert et al., 1993), onion center rot (*Allium cepa*) (Stumpf et al., 2018; Koirala et al., 2021), leaf blight on maize (Mangeli et al., 2024), and rice (*Oryza sativa*) (Azizi et al., 2019). Diseases caused by phytopathogenic *Pantoea* isolates are diverse (Walterson and Stavrinides, 2015) and are facilitated by multiple pathogenicity factors, including the type III secretion system (T3SS), quorum-sensing systems, and exopolysaccharides (EPS) (Beck Von Bodman and Farrand, 1995; Von Bodman et al., 1998; Roper, 2011). The T3SS is a complex extracellular injection apparatus forming channels that cross the bacterial envelope and the host cell membrane, deploying ATP and chaperonins to translocate type III effectors (T3Es) into the interior of nearby host cells, resulting in the subversion of host defenses (Galán and Collmer, 1999). However, in non-host or resistant plants, the recognition of T3Es or their subversive activities can elicit a second wave of defenses, including HR (Deng et al., 2017). In plant-pathogenic bacteria, the *hrp* (HR and pathogenicity) locus encodes a T3SS and effectors.

In *Pnss* DC283, three T3SSs are encoded on distinct *Pantoea* secretion islands (PSI-1 to PSI-3) (Kirzinger et al., 2015). *Pnss* DC283 uses two T3SSs, located on two separate megaplasmids (Duong et al., 2017), to colonize its two hosts, one for corn and the other for the beetle (Correa et al., 2012). *Pnss* colonizes maize leaf apoplast and xylem, where it proliferates and causes Stewart’s wilt and leaf blight, respectively (Roper, 2011). In maize, the pathogenesis is mediated by a plant-specific T3SS, PSI-1 (Hrc-Hrp 1) (Correa et al., 2012), a conserved 24 kb genome island that produces needle-like injectisomes (pili) that inject an effector WtsE into apoplast cells, causing water-soaked lesions in the first phase (Ham et al., 2006; Correa et al., 2012). The Hrc-Hrp1 family of T3SSs is widespread among other enterobacterial plant pathogens, including *Erwinia*, *Dickeya*, and *Pectobacterium* spp. (Frederick et al., 2001; Correa et al., 2012; Kirzinger et al., 2015), and it is also found in *P. agglomerans* pv. *gypsophilae* (Moretti et al., 2021). Expression of this system was upregulated during *in planta* growth relative to *in vitro* growth (Packard et al., 2017). Mutations of either the T3SS or just WtsE cause substantial reductions in *Pnss* proliferation, inducing water-soaked lesions in the apoplast of infected maize leaves (Ham et al., 2006). *Pnss* DC283 harbors PSI-2, which is orthologous to the Inv-Mxi-Spa T3SS family (SPI-1) found in pathogens such as *Salmonella* (Lou et al., 2019). This enables persistence and colonization within the corn flea beetle vector (Correa et al., 2012). *P. stewartii* subsp. *indologenes* pv. *cepacicola* pathogenicity on millet is also T3SS-dependent (Koirala et al., 2021). Either the HiVir or halophos phosphonate biosynthetic gene cluster was required for onion necrosis phenotype virulence (Zhao et al., 2023). While *Pnss* is a T3SS-dependent pathogen with relatively explicit pathogenic mechanisms, the pathogenicity determinants of *Pnsi* remain unclear.

Bacterial flagella are essential for motility (Chevance and Hughes, 2008). Approximately 45% of annotated flagellar systems have more than one flagellin gene essential for filament assembly, motility, adhesion, and cytotoxicity (Faulds-Pain et al., 2011; Hu and Reeves, 2020). In the *Pnss* DC283, surface motility is a critical component of disease development, and this trait is flagella-dependent (Herrera et al., 2008). A rice colonizer endophytic *Azoarcus* sp. strain BH72 harbors three copies of the *fliC* gene; only *fliC3* was highly expressed and required for motility but not for elicitation of defense responses as a microbe-associated molecular pattern (MAMP) (Buschart et al., 2012). Phytopathogens with multiple flagellins are a key factor associated with full virulence, facilitating colonization and plant immune responses. In *Pseudomonas aeruginosa*, flagellar assembly and/or mobility antagonize the T3SS with a negative crosstalk between these two systems (Soscia et al., 2007), indicating that some virulence factors are coordinated but inversely regulated.

Plants and animals recognize conserved, widely distributed MAMPs via diverse extracellular surface-localized pattern recognition receptors (PRRs) and stimulate MAMP-triggered immunity (MTI) to restrict microbe growth (Teixeira et al., 2019; Zhou and Zhang, 2020). The best-characterized MAMP is flg22, an epitope from the N-terminus of flagellin (Felix et al., 1999; Boutrot and Zipfel, 2017). Flg22 is required for bacterial motility and pivotal for activating MTI responses in substantial interrogations (Gómez-Gómez et al., 1999; Naito et al., 2008). The short flagellin peptides require plant hydrolytic release from the flagellin precursor before they are perceived by the plant PRR FLAGELLIN SENSING2 (FLS2) (Buscaill et al., 2019). The flg22 epitope binds FLS2 and recruits a co-receptor, BRI1-ASSOCIATED KINASE 1 (BAK1) and determines the formation of the flg22-FLS2-BAK1 complex (Chinchilla et al., 2007; Sun et al., 2013). This initiated MTI responses, including ROS burst and seedling growth inhibition in *Arabidopsis thaliana* (Kadota et al., 2014). Strong selective pressure from the plant immune system has driven bacterial flagellin to evolve strategies to evade and suppress these immune responses, most directly via epitope modification (Buscaill and Van Der Hoorn, 2021). Two aspartic residues (Asp^14^ and Asp^15^) in the flg22 define a dual signature for FLS2 recognition and bacterial motility (Sun et al., 2006; Wang et al., 2015; Parys et al., 2021). The I21A polymorphism of flg22^Rso^ from *Ralstonia solanacearum* avoids perception by AtFLS2, along with Gly^18^, which is important for BAK1 binding, as well as Leu^19^ and Gln^20^, which form a GLQ hydrophobic patch to stabilize the binding of FLS2-BAK1 (Sun et al., 2013; Wei et al., 2020).

*Pantoea stewartii* is a remarkable bacterial system for laboratory studies because of its relative ease of propagation and genetic manipulation and its likely minimal number of pathogenicity mechanisms. Therefore, studies on multiple flagellins have mostly focused on a handful of pathogens and beneficial bacteria associated with certain plants (Colaianni et al., 2021). However, the roles of multiple flagellins in rice-associated *P. stewartii* subsp. *indologenes* remain poorly characterized. Here, we test the hypothesis that *Pnsi* HR3-48 uses a multifaceted virulence strategy. The conserved PSI-1 T3SS primarily promotes virulence by modulating host immunity, whereas the flag-1 *fliC* repertoire supports motility and sets the magnitude of MTI activation. We further examine the residue-19 variation in flg22-tune immune visibility across hosts. To this end, we combine comparative genomics with targeted mutants to link these modules to plant phenotypes. The findings deepen our understanding of the pathogenesis of rice-associated *Pnsi* and of how pathogens navigate plant immunity through the evolution and regulation of the virulence apparatus.

## Materials and methods

### Strains, plasmids, and plant materials

*Escherichia coli* and *P. stewartii* derivative strains were grown at 37 °C and 30 °C, respectively, in LB broth or on 1.2% LB agar plates. The growth medium was supplemented with chloramphenicol (Cm; 25 μg/mL), kanamycin (Km; 50 μg/mL), spectinomycin (Sp; 50 μg/mL), and apramycin (Apr; 50 μg/mL) (Supplementary Table S2). *N*. *benthamiana* was grown in a greenhouse with 14 h light (20,000 Lux) at 24 °C and 10 h dark at 22 °C and 65% relative humidity. *A. thaliana* plants were grown under 16 h day/8 h night conditions at 22 °C and 70% relative humidity. ‘Jubilee’ maize seedlings were planted and grown for 8 days in a phytotron at 28 °C, 80% relative humidity, 16 h light and 8 h dark cycles, and a light intensity of at least 200 mE m^−2^ s^−1^. JG30 and Dular rice plants were planted in the screenhouse at the Institute of Crop Science, Chinese Academy of Agricultural Sciences, Beijing, China.

### Whole-genome dataset preparation

A total of 41 whole genomes with complete genome assembly levels, comprising *Pantoea* and *E. coli* strains, were retrieved from GenBank. Sequence similarity analysis was performed using the 16S rRNA gene sequence (1,542 bp) extracted from HR3-48 (GCF_025765915.1) on the EzBioCloud website^1^. A total of 22 *Pantoea* species were selected for downstream analyses, including 20 closely related *Pantoea* species and 21 *P. stewartii* strains. Whole-genome sequencing was performed with ONT (Nanopore) and analyzed by Biomarker Biotechnology Co., Ltd. (Beijing, China). Supplementary Table S1 shows strain isolation sources from the NCBI BioSample database and other strain metadata.

### Phylogenetic analyses

A maximum-likelihood (ML) phylogeny of the 42 *Pantoea* strains was constructed using the UBCG tool (v3.0) based on 92 conserved housekeeping genes (Na et al., 2018). Tree reconstruction was performed with FastTree (Price et al., 2010). Phylogenies were visualized using the interactive online tool iTOL (Letunic and Bork, 2024), and evolutionary relationships were compared.

### Average nucleotide identity and DNA–DNA hybridization analysis

To clarify species affiliations, average nucleotide identity (ANI) and digital DNA–DNA hybridization (dDDH) were used to assess nucleotide-level similarity for all pairwise genome combinations. Pairwise ANI values were calculated using FastANI (v1.33) with default settings (Jain et al., 2018). All pairwise dDDH values were calculated using GGDC 2.1^2^ with the recommended Formula 2, using BLAST for sequence alignment. For parallel visualization of dDDH and ANI data, plots were generated using ggplot2 in R version 4.5.1.

### Comparative genomic analyses of *Pantoea stewartii* T3SS cluster and flagellar loci

Seven previously characterized *Pantoea* complete genomes and one newly sequenced strain were examined to compare T3SS loci and the flagellar gene cluster *flag-1*. T3SS identification was guided by literature-defined architectures for three modules, Hrp-1 (plant-associated Hrc/Hrp), SPI-1, and SPI-2. Conserved orthologues were used to query each genome with BLAST+ (blastp/tBLASTn) against the NCBI non-redundant (nr) protein database and to delimit cluster boundaries by synteny and flanking-gene context (Hrp1: *hrcC*, *hrcN*, *hrcU*, *hrcV*; SPI-1: *invG*, *spaL*, *spaS*, *invA*, *prgH/I/J/K*; SPI-2: *ssaC*, *ssaN*, *ssaU*, *ssaV*, and *ssrA/ssrB*). Canonical SPI-1/SPI-2 organization was cross-checked against *Salmonella enterica* to verify operon content and order. Hrp-1 assignments followed the *hrp/hrc* gene complements of typical plant-associated systems.

The flagellar region was delineated by locating the contiguous *flh–flg–fli* operon and *fliC* homologs. Genome-wide surveys for dispersed *fliC* copies combined BLASTP searches seeded with FliC sequences using PFAM annotations of flagellin-N (pfam00669) and flagellin-C (pfam00700) motifs. For each dispersed *fliC* locus, adjacent insertion sequence/transposase (IS/Tnp) features were annotated. The linear distances to the nearest upstream and downstream IS/Tnp were calculated from GFF coordinates.

### Identification of putative T3Es

To investigate putative type III effectors (T3Es), we conducted procedures as previously reported. First, all explicitly known *P. stewartii* T3Es, harpins, chaperones, and secretion-associated proteins that had been reported were manually extracted, and TBLASTN was used against the HR3-48 genome to identify orthologs, with an E-value cutoff of 1 × 10^−5^. Sequence coverage, identity, annotation, and genomic context were subsequently examined for orthology assignment. Independently, a Hidden Markov model (HMM) was built using known *hrp-box* motifs (xGGAACx[N_15–16_]CCACxxAG) (Yang et al., 2010) reported previously with HMMER (v3.3.2) (Potter et al., 2018). These motif sequences were aligned using MAFFT, and the HMM was constructed using hmmbuild. HR3-48 genome was scanned using the *hrp-box* HMM model. Genes located within 10 ORFs downstream of a predicted *hrp-box* motif and carrying a predicted N-terminal T3SS secretion signal using EffectiveT3 (v1.0.1) with a selective parameter setting (Jehl et al., 2011) were retained as T3SS-associated candidates. Candidates were further evaluated by their location within the PSI-1 region, absence of predicted transmembrane or Sec-dependent signal peptides, and eggNOG/Pfam annotation. Proteins with effector-family homology were designated putative T3Es, whereas harpins, chaperones, and translocon-associated proteins are classified separately in Supplementary Table S6.

### CRISPR-Cas9-mediated gene editing and HR recombination-mediated construction of *fliC* mutants

Plasmids and primers are listed in Supplementary Table S3. To our knowledge, the CRISPR-Cas9-mediated gene editing system was one of the first applications used in *Pantoea*. The toxicity of *Streptococcus pyogenes cas9* (SpCas9) was detected before we used the editing system. The crRNA, containing 20 oligonucleotides of the target gene^3^ or manually selected 20 nt in a distinct region of the *fliC* sequences with high identity (> 90% in general), was amplified using primers (Supplementary Table S3). The amplification products carrying overhang ends were fused and ligated into the ApaLI/NsiI-digested pRK2-CRISPR to generate pRK2-CRISPR-HR3-48*fliC*s-sgRNA. For the pRK2-CRISPR-HR3-48*fliC*s*-*donor construction, approximately ~1 kb upstream and ~1 kb downstream homology arms (HAs) flanking *fliC*s were amplified using a seamless cloning kit. The transformation was performed by triparental mating with helper pRK600 to introduce the plasmid pRK2-CRISPR-HR3-48*fliC*s*-*donor into HR3-48, and mixtures were incubated at 30 °C for 48 h. The lawn was diluted and spread on selective plates containing 50 μg/mL apramycin and 25 μg/mL spectinomycin for 24–48 h at 30 °C. The primers (Supplementary Table S3) were used to verify the mutants, and then they were sequenced them at Tsingke Biotechnology Co., LTD. To recover the correct editing plasmid, a mutant colony harboring the donor plasmid was inoculated in LB liquid medium with 10% sucrose for 24 h. The culture was then spread on LB agar plates with 10% sucrose for 18–24 h. A single colony was inoculated by stabbing into the LB plate with spectinomycin plus 10% sucrose and 25 μg/mL spectinomycin plus 50 μg/mL apramycin. Colonies that grew only on the former plates but not the latter were confirmed as plasmid-free by colony PCR with primers Apr-F/R or by examining apramycin sensitivity.

*fliC1, fliC4,* and *fliC5* exhibit a high nucleotide identity (93.72%), making it difficult to mutate the three genes separately with a suitable sgRNA. Therefore, we used the pK18mobsacB-mediated HR system for mutations. Transformants of pK18mobsacB harboring the pMB1 *ori* could replicate in HR3-48, which obscures the first crossover. Therefore, we replaced the pMB1 *ori* with the R6K *γ ori* amplified from pCPP6529 to create pK18mobsacB (R6K). This plasmid replicates only in π protein-producing hosts (Chen et al., 1998), whereas HR3-48 does not produce π protein. Upstream and downstream HAs of *fliC1* were cloned into SmaI/BamHI-digested pK18mobsacB (R6K) to generate pK18mobsacB-HR3-48*fliC1*. Constructs were transformed into *E. coli* DH5α λ *pir* on LB agar medium containing kanamycin, X-Gal, and IPTG; verified by M13F/R primers and conjugated into HR3-48 by triparental mating. The lawn samples were plated onto LB agar medium with kanamycin (Km^r^) and spectinomycin (Sp^r^) and incubated overnight. Km^r^ Sp^r^ transformants were passed once in antibiotic-free LB for 24 h and counter-selected on spectinomycin plus 10% sucrose. Colonies were randomly selected and stabbed to propagate in LB medium with Km^r^ Sp^r^ and spectinomycin plus 10% sucrose at 30 °C overnight. Colonies that grew on spectinomycin plus 10% sucrose medium plate but not Km^r^ Sp^r^ plate were selected for PCR amplification with the HR3-48Δ*fliC1*-F/R primer pair (Supplementary Table S3) and confirmed by sequencing to contain in-frame deletions.

### Motility assay

Motility assays were conducted on LB medium with 0.2% (w/v) agar for swimming or 0.3% (w/v) agar supplemented with 0.5% glucose for swarming. Strains were streaked on LB plates and incubated overnight. For motility assays, a single colony was picked using a sterile toothpick and inoculated at the center of LB agar plates. Plates were incubated at 30 °C for 24 h (swimming) or 48 h (swarming). Motility halos were photographed and quantified using ImageJ by measuring the distance from the inoculation point to the edge of visible bacterial spread. All the experiments were performed in triplicate.

### Transmission electron microscopy observation

Wild-type strains and mutants were subjected to negative staining (Mukhitov et al., 2016). Fresh bacterial colonies in the logarithmic growth phase were washed twice with sterile PBS and adjusted to OD_600_ = 0.5. Copper grids (200-mesh) with a carbon film were pretreated with a plasma cleaner for 30 s to improve hydrophilicity. Then 5 μL of the bacterial suspension was applied to the grid for 1 min, followed by 5 μL of 1% uranyl acetate for staining at 28 °C for 30–60 s. Excess stain was removed with filter paper, and grids were air-dried for 10 min. Samples were imaged using a Hitachi HT7700 Exalens (Hitachi, Tokyo, Japan) at the Institute of Crop Science, Chinese Academy of Agricultural Sciences (Beijing, China).

### qRT-PCR for analysis of *fliC* expression

*Pantoea* strain HR3-48 cultures were grown to mid-exponential phase (OD₆₀₀ ≈ 0.6), harvested, and total RNA was extracted using the E.Z.N.A.® Bacterial RNA Kit (Omega, Cat. No. R6950-02) according to the manufacturer’s instructions. cDNA was synthesized from 200 to 500 ng of RNA using the Evo *M-MLV* RT Mix Kit with gDNA Clean for qPCR (Accurate Biology, Code No. AG11728). The cDNA was diluted 10-fold, and 2 μL was used in 20 μL qPCR reactions containing SYBR® Green Premix Pro Taq HS (Accurate Biology, Code No. AG11701) and 0.2 μM of each primer, with *gyrB* as the normalization control. Relative expression levels were calculated using the 2^–ΔCt^ method. All primers are listed in Supplementary Table S3.

### ROS assay of bacterial and peptide flg22 in *Nicotiana benthamiana* and *Arabidopsis thaliana*

ROS production induced by the *Pantoea* strains was measured following the method (Wei et al., 2013). HR3-48 and its derivatives were infiltrated into 5-week-old *N. benthamiana* leaves at 1 × 10^8^ cfu/mL. At 6 h post-infiltration, 12 leaf disks were pressed gently by the 5 mm diameter biopsy punch, collected in a 96-well microplate containing 100 μL ddH_2_O, and then 100 μL of 0.5 mM L-012 (Wako, Japan) in 10 mM MOPS–KOH buffer (pH 7.4) was added. The ROS generation intensity was determined by monitoring the chemiluminescence using a luminometer (Infinite M200PRO, Tecan) for 6 h. A solution of 10 mM MgCl_2_ was infiltrated as the negative control.

For detection of ROS burst induced by peptide flg22 in *N*. *benthamiana* and *A. thaliana*, we used a luminol-based chemiluminescence assay following Sang and Macho (2017) manual instructions and Colaianni et al. (2021). Four-week-old *N. benthamiana* leaf discs were collected and placed adaxial side up in a white 96-well microplate with 200 μL sterile distilled water in a controlled environment room for 14–16 h to reduce wound-associated background signals. After removing ddH_2_O, a 200 μL elicitor master mix [100 nM flg22, 100 μM luminol, 20 μg/mL horseradish peroxidase (HRP)] was added to each well, and ROS production was measured immediately over 60 min. *A. thaliana* plants were grown under 16 h day/8 h night conditions for 4 weeks. Leaf discs were collected from fully expanded rosette leaves using a 5-mm biopsy punch and floated overnight in sterile distilled water in white 96-well plates. The following steps were the same as for *N. benthamiana* mentioned above. Mock treatment contained the reaction solution without peptide. Luminescence was recorded immediately using a microplate luminometer for 60 min. ROS production was presented as relative light units (RLU) over time. At least 12 leaf discs from three independent plants were used per treatment, and the experiment was independently repeated three times.

### ROS measurement of peptide flg22 in rice cultivar Dular

ROS production in rice (Dular) seedlings was quantified using a luminol-based chemiluminescence assay, with minor modifications to a previously described method (You et al., 2023). Avoiding the vein, leaf disks were excised from the second leaves of 5-week-old plants grown in the greenhouse at 30 °C. Three disks were immersed in a 1.5 mL microcentrifuge tube (Axygen) with 100 μL of sterile distilled water overnight to recover from wound injury. Leaf disks were then transferred into 100 μL of luminol containing 1.0 μL of horseradish peroxidase (1 μg/μL) from Bio-Rad (Catalog no. 170–5,040), and elicitor (1 μM flg22 peptide or sterile water as a control). Chemiluminescence was measured using a GloMax 20/20 luminometer at 1/10s for 25 min. Three independent biological replicates were analyzed, and three technical replicates were included for each sample, with at least three leaf disks per treatment.

### Challenge-inoculation hypersensitive response (HR) of bacteria and flg22

Primary streak cultures of *Pantoea* strain HR3-48, its derivatives, and *P. syringae* pv. *tomato* DC3000 (*Pst* DC3000) were grown overnight on LB and KB agar plates with antibiotics. To test the ability of HR3-48 and its derivatives on the associated inhibition of the HR, HR3-48 and its derivatives were resuspended in 10 mM MgCl_2_, adjusted to a concentration gradient at 1 × 10^8^ cfu/mL and 5 × 10^7^ cfu/mL, then infiltrated into at least six leaves from two 6-week-old *N. benthamiana* plants. Six hours post-infiltration, the leaf areas were challenge-inoculated with 1 × 10^7^ cfu/mL *Pst* DC3000, half overlapping the pretreated infiltration sites. Leaves inoculated with *Pst* DC3000 alone consistently exhibited HR. Meanwhile, areas treated with 10 mM MgCl_2_ served as baseline controls. All assays were conducted in triplicate, and leaves were photographed 48 h post-challenge. A measure of 1 μM flg22 epitopes was infiltrated into young 4-week-old *N. benthamiana* plants to suppress HR following the protocol described above. Each experiment was repeated as indicated.

### Maize and rice virulence assays

Bacterial cells were cultured in LB medium supplemented with 0.2% glucose until reaching OD₆₀₀ = 0.5, then harvested by centrifugation for 2 min at 6,500 rpm. The cell pellet was washed once and resuspended in 1 × PBS containing 0.2% Tween 40. Average-sized healthy seedling leaves of 8-d-old sweetcorn (Hybrid Jubilee) were scratched with a sterilized toothpick to form a 1 cm^2^ square area halfway along the expanding leaf. Approximately 5 × 10^8^ cfu/mL of bacterial culture were pipetted into the scratch area. Inoculated plants were maintained in a growth chamber, and disease progression was evaluated at 14-day post-inoculation (dpi) using the disease rating scale (Michener et al., 2003).

For rice inoculation, 4-week-old JG30 rice seedlings were inoculated with bacterial suspension at 1 × 10^9^ cfu/mL per strain using leaf clipping. Sterile scissors were used to soak the bacterial solution, and the tips of 2 cm of fully expanded rice leaves were cut, with three leaves per plant and three plants each for phenotypic identification. Inoculated seedlings were maintained in a growth chamber at 26–35 °C. Disease development was monitored daily, and disease severity was assessed by measuring lesion length, defined as the distance from the inoculation site to the edge of fully developed blighted tissue along the midvein of the leaf, and photographed after 2 weeks (Zhang et al., 2013; Ke et al., 2017).

### Dular rice germination rate

Full-grain seeds were baked at 40–45 °C for 2–3 days, surface-sterilized with 2–3% sodium hypochlorite for 30 min and 75% ethyl alcohol for 5 min, followed by washing 7–8 times with sterile water, and then incubated at 37 °C to break dormancy. Petri dishes containing 1 × Murashige and Skoog (MS) medium supplemented with 0.5% sucrose and adjusted to pH = 5.8 with KOH were prepared. Ten treated seeds were placed on each plate. Seeds were submerged in bacterial suspensions of HR3-48 and its derivatives at a gradient dilution of 1 × 10^6^ cfu/mL and 1 × 10^5^ cfu/mL for 30 min. A 10 mM MgCl_2_ solution was used as a negative control. Plates were incubated at 28 °C, and germination rates were recorded and photographed at 3–5 days. Each treatment was performed in triplicate, and the experiment was repeated three times.

### Seedling growth inhibition (SGI) assay

*Arabidopsis thaliana* Col-0 seeds were surface sterilized in 0.2% bleach for 10 min, followed by 75% ethanol for 2 min, and rinsed 7–8 times with sterile distilled water (Gómez-Gómez et al., 1999). Seeds were germinated on ½ MS plates containing 0.8% plant agar and 1% sucrose and stratified at 4 °C in the dark for 48 h before being grown under 16 h light/8 h dark, 21 °C day/23 °C night, and 60% relative humidity. Five-day-old average-sized seedlings were transferred to 35 mm tissue culture dishes containing 3 mL of liquid ½ MS medium supplemented with 1 μM flg22 peptide and 10 seedlings per dish. After 10 days, a minimum of eight seedlings per treatment were blotted dry and weighed. Negative controls (no peptide) and positive controls flg22^Pae^ were included in each experiment.

## Results

### Phylogenetic and HR3-48 genomic analyses

Whole-genome sequencing of HR3-48 showed a single circular chromosome (4,439,845 bp) and one plasmid (333,832 bp), with 53.88% G + C content (Supplementary Figure S1A). The genome encodes 4,357 predicted protein-coding genes (average 944 bp) annotated with eggNOG (an updated version of COG) (Supplementary Figure S1B), Gene Ontology (Supplementary Figure S1C), and KEGG using BLAST v2.2.29 (Supplementary Figure S1D). We analyzed the complete 16S rRNA gene sequence (1,542 bp) using EzBioCloud (Chalita et al., 2024), yielding 22 closely related *Pantoea* species, among which the *P. stewartii* group had the highest similarity. The closest matches were *P. stewartii* subsp. *indologenes* LMG 2632^T^ (99.38% similarity), *P. brenneri* LMG 5343^T^ (98.29%), and *P. ananatis* LMG 2665^T^ (98.22%). Only LMG 2632^T^ exceeded 98.7% for species identity (Riesco and Trujillo, 2024).

To confirm the phylogenetic relationships, we built a maximum-likelihood phylogeny from the concatenated Up-to-date Bacterial Core Gene (UBCG) consisting of 92 housekeeping genes (Na et al., 2018). HR3-48 clustered with *P. stewartii* subsp. *indologenes* LMG 2632^T^ (bootstrap 100%), distinct from other *Pantoea* type strains (Supplementary Figure S2A). Taxonomic identification was performed by the pairwise digital DNA–DNA hybridization (dDDH) and average nucleotide identity (ANI) values compared with other *Pantoea* spp. type strains. The highest ANI values were *P. stewartii* subsp. *indologenes* PNA 15–2 (99.09%), *P. stewartii* subsp. *indologenes* NCPPB 2281 (99.07%) and *P. stewartii* subsp. *indologenes* LMG 2671 (99.07%) (Supplementary Figure S2B; Supplementary Table S4). The highest dDDH value was *P. stewartii* subsp. *indologenes* LMG 2632^T^ (96.95%) (Supplementary Figure S2B; Supplementary Table S5). The type strains of *P. stewartii* subsp. *indologenes* (LMG 2632^T^) and *P. stewartii* subsp. *stewartii* (ATCC 8199^T^) and other *P. stewartii* strains shared a dDDH value > 96% and ANI values > 98% with HR3-48. However, HR3-48 showed ANI < 85% and dDDH values < 62% with *P. ananatis* LMG 2665^T^, *P. allii* LMG 24248^T^, and other type strains below the species cut-off values (70% for dDDH, 95 to 96% for ANI) (Richter and Rosselló-Móra, 2009). In conclusion, HR3-48 represents *P. stewartii* subsp. *indologenes*.

### Comparing T3SSs and flagellar loci in *Pantoea stewartii*

We compared the T3SS and flagellar locus of the *P. stewartii* subsp. *indologenes* HR3-48 with six other complete *P. stewartii* strains using *S. enterica* as a comparison. *P. stewartii* carries phylogenetically distinct T3SSs. Only the *Pnss* DC283 had three types of T3SS: a 29-kb PSI-1 gene cluster on one plasmid, a 26-kb PSI-2, and a 25-kb PSI-3 gene cluster on another plasmid (Figure 1A), previously associated with maize pathogenesis (Correa et al., 2012). *Pnss* ATCC 8199^T^ retained PSI-3 of unknown function, which was extensively conserved with SPI-2 of *Salmonella* and *Yersinia* (Büttner, 2012). Meanwhile, all *Pnsi* strains only harbored a plant cell-specific PSI-1 (Figure 1A). *P. stewartii* PSCN1, isolated from sugarcane with bacterial leaf wilt symptoms (Cui et al., 2021), and *P. stewartii* subsp. *indologenes* RON18713, isolated from the Brazil nut tree phyllosphere (Rocha et al., 2023), lacked any T3SS. All PSI-1 (Hrc-Hrp 1) comprised approximately 28 proteins, including *hrc* open reading frames (ORFs) for the secretion machine, *hrp* operons for regulatory proteins, and variable secreted effectors, including *wtsE*. Genomic composition and organization were similar across strains with the *wtsE-wtsF* cassette, indicating that the PSI-1 structure was conserved in *P. stewartii* (Figure 1A). Only *Pnss* DC283 had the effector HopAM1 that aids pathogen adaptation to water availability (Goel et al., 2008; Gentzel et al., 2022), as a truncated version of two fragments (Figure 1A). PSI-1 contains the conserved *wtsE–wtsF* cassette and the harpin gene *hrpN* (Figure 1A). Previous studies in *P. stewartii* subsp. *stewartii* showed that WtsE is a major virulence determinant for corn pathogenicity, whereas HrpN is dispensable for pathogenicity (Ham et al., 2006, 2008; Roper, 2011). However, the functional role of *hrpN* in *Pnsi* remains untested. Five high-confidence (E-value ≤ 1 × 10^−5^) T3SS-associated candidates were retained in HR3-48 (Supplementary Table S6), including two putative T3Es, AvrE-family protein WtsE and an AvrB-family protein WtsB that have been reported previously (Ham et al., 2006; Zhao et al., 2023), together with the harpin HrpN (Ahmad et al., 2001) and the T3SS-associated translocon HrpF (Frederick et al., 2001). Orthology comparison identified WtsE as the conserved DC283 effector (WtsE, ARF52487.1) homolog (99.02%), whereas the WtsB was absent from the characterized DC283 effector repertoire but found in *Pnsi* PNA 03-3 (AvrB3, PXV73943.1) (Zhao et al., 2023) with 100% identity (Supplementary Table S6), providing a focused resource for prioritizing follow-up effector testing in *P. stewartii* subsp. *indologenes*. Notably, we did not experimentally test their secretion and translocation. Diverse transposase families and tRNAs were identified flanking PSI-2 and PSI-3 (Figure 1A), consistent with prior evidence for vertical inheritance of PSI-1 and ancient or recent horizontal acquisition of PSI-2 and PSI-3 (Kirzinger et al., 2015).

**Figure 1:**
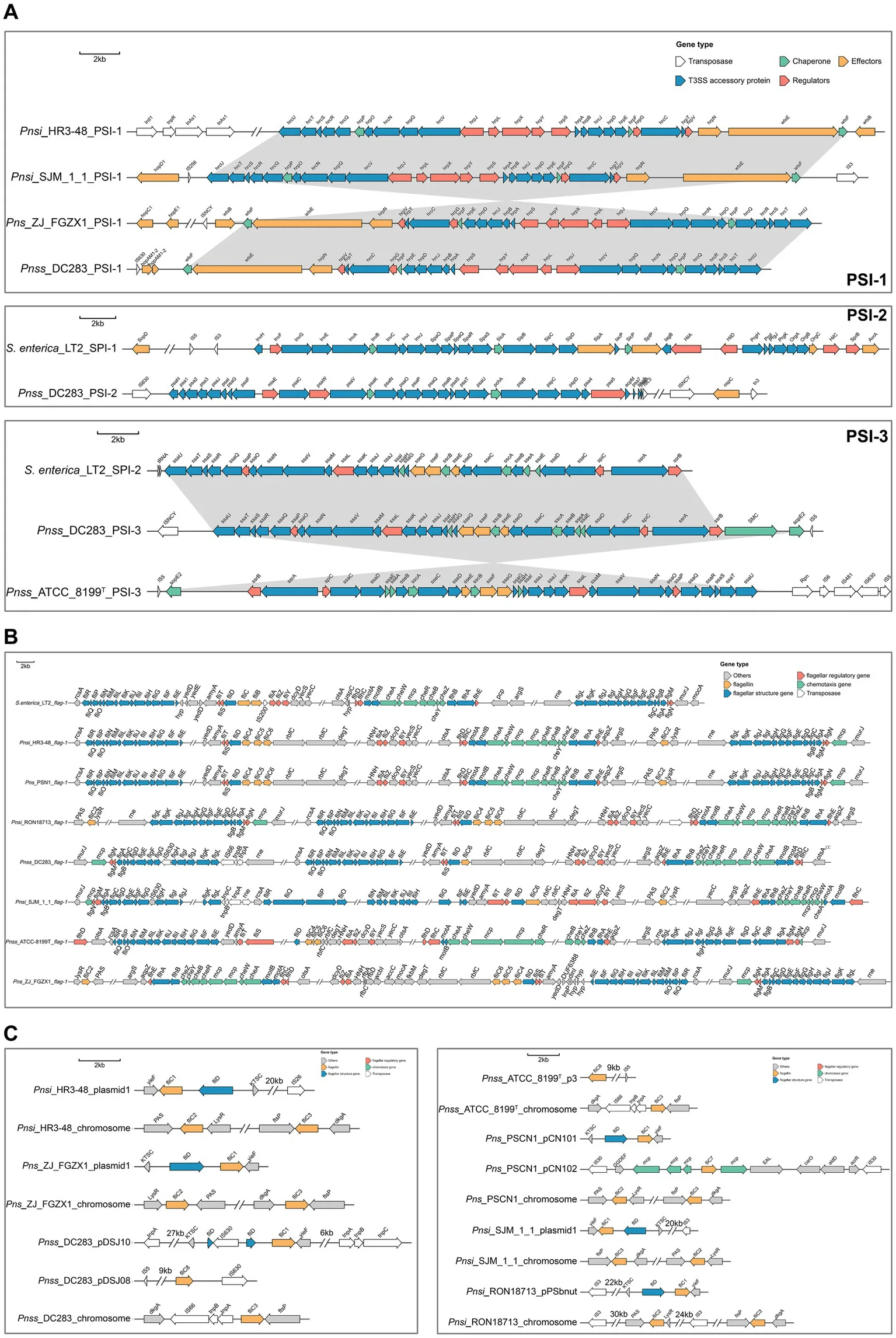
Comparison of genetic organization of T3SS and flagellar locus (*flag-1*) among seven *P. stewartii* species. **(A)** Arrow colors symbolize specific gene composition and varied functional proteins within T3SS. Gene names are provided at the top of the linear graph. Pairwise alignment between linear sequences was generated using the BLAST algorithm with cut-off values from 65 to 100%. Regions with higher nucleotide identity are displayed in shaded grey. Double slashes at genome islands indicate a sequence interval. *S. enterica* subsp. *enterica* LT2 with SPI-1 and SPI-2 T3SS was used for comparison. SPI-1, *Salmonella* pathogenicity island 1, SPI-2, *Salmonella* pathogenicity island 2, *hrc* (hypersensitive response and pathogenicity conserved genes), *hrp* (hypersensitive response and pathogenicity or hairpin proteins), *hop* (*hrp* outer protein), *wtsE* (water-soaked effector protein E), *wtsF* (water-soaked effector protein F), *wtsB* (water-soaked effector protein B). Other proteins in PSI-2 and PSI-3 were orthologues of PSI-1, which were proposed with species-specific names (Deng et al., 2017). **(B)** Schematic comparison of the *flag-1* loci of 7 complete genomes within *P. stewartii* and comparison with clusters of *S. enterica* subsp. *enterica* LT2. Flanking and flagellar-associated genes are colored in light grey. Others are shown as indicated in the legend. **(C)** Schematic diagram of scattered flagellin (*fliC*) gene clusters in different positions of chromosomes and plasmids, with their adjacent mobile genetic elements, including IS family genes and transposases (Tnp), indicated with blank arrows. Genomic distance was also calculated. A scale bar (2 kilobases) indicates the size of all loci.

TBlastN of seven complete *P. stewartii* genomes showed a single flagellar locus (*flag-1*), with three major genomic clusters on average 25 kb encoding approximately 50 proteins, including block 1: *fliRQPONMLKJIHGFE-fliTSDC-fliAZY*, involved in regulation and assembly of the basal body, filament (*fliC*), and σ^28^ factor *fliA*; block 2: *flhDC-motABcheAWRBYZ-flhBAE*, encoding master regulator (*flhDC*) and motor proteins (*motAB*); block 3: *flgLKJIHGFEDCBAMN*, encoding flagellar structural proteins (Soutourina and Bertin, 2003). Gene block order varies among strains to assemble an intact flagellum (Figure 1B). We identified 3–7 *fliC* genes per strain with high amino acid identity (90.62%, Supplementary Figure S3A; Supplementary Table S1). Five of seven strains had a tandem *fliC* triplet (*fliC456*) within *flag-1*, with additional copies on the chromosomal regions outside the main flagellar locus *flag-1* or on plasmids (Figures 1B, C). Scanning 38 FliC proteins of seven *P*. *stewartii* strains identified 3 unique flg22 epitopes, and only represented variants at the 19th residue (L19E, L19Q, and L19M) (Supplementary Table S1). Mobile genetic elements flanking *fliCs* genes, except for *flag-1* (Figure 1C), indicated that recombination and horizontal gene transfer (HGT) might have increased flg22 polymorphisms at Glu^19^.

### CRISPR-Cas9-mediated gene editing and homologous recombination of *fliCs* in HR3-48

To investigate flagellin function, we generated in-frame deletions of *fliC* genes in HR3-48 using CRISPR-Cas9 and pK18mobsacB. Conjugation with pHC60, pRK2-CRISPR, pRK2-CRISPR-HR3-48*fliC1*-sgRNA, and pRK2-CRISPR-HR3-48*fliC2*-sgRNA yielded transformants at 3.08 × 10^−2^, 1.67 × 10^−2^, 3.9 × 10^−5^, and 2.5 × 10^−7^ cells per recipient (Figure 2A). The cleavage frequency of pRK2-CRISPR-HR3-48*fliC1*-sgRNA was > 1,000-fold lower than that of pRK2-CRISPR with SpCas9, whose efficiency was close to controls. This indicated that SpCas9 was non-lethal to HR3-48 but unsuitable for HR3-48*fliC1* knockout. Conjugation with pRK2-CRISPR harboring crRNA of HR3-48*fliC3*, HR3-48*fliC6,* and HR3-48*fliC456* was four, four, and five orders of magnitude lower than in the controls (Figure 2A). This showed that SpCas9, guided by efficient sgRNA, could induce DSB in HR3-48. PCR amplification and DNA region sequencing of the target site confirmed *fliC*s in-frame deletion (Figures 2C,D,G,H). Due to the high nucleotide sequence identity among *fliC1*, *fliC4,* and *fliC5* (94.02%), we used a suicide plasmid pK18mobsacB-mediated seamless genome editing to knock out these *fliC* genes. Although the efficiency of *fliC1*, *fliC4,* and *fliC5* deletion was equal to that of CRISPR-Cas9-mediated gene editing (Figures 2B,E,F), the construction period was shortened by 2 days, suggesting that CRISPR-Cas9 has great potential for *Pantoea* genetic manipulation.

**Figure 2:**
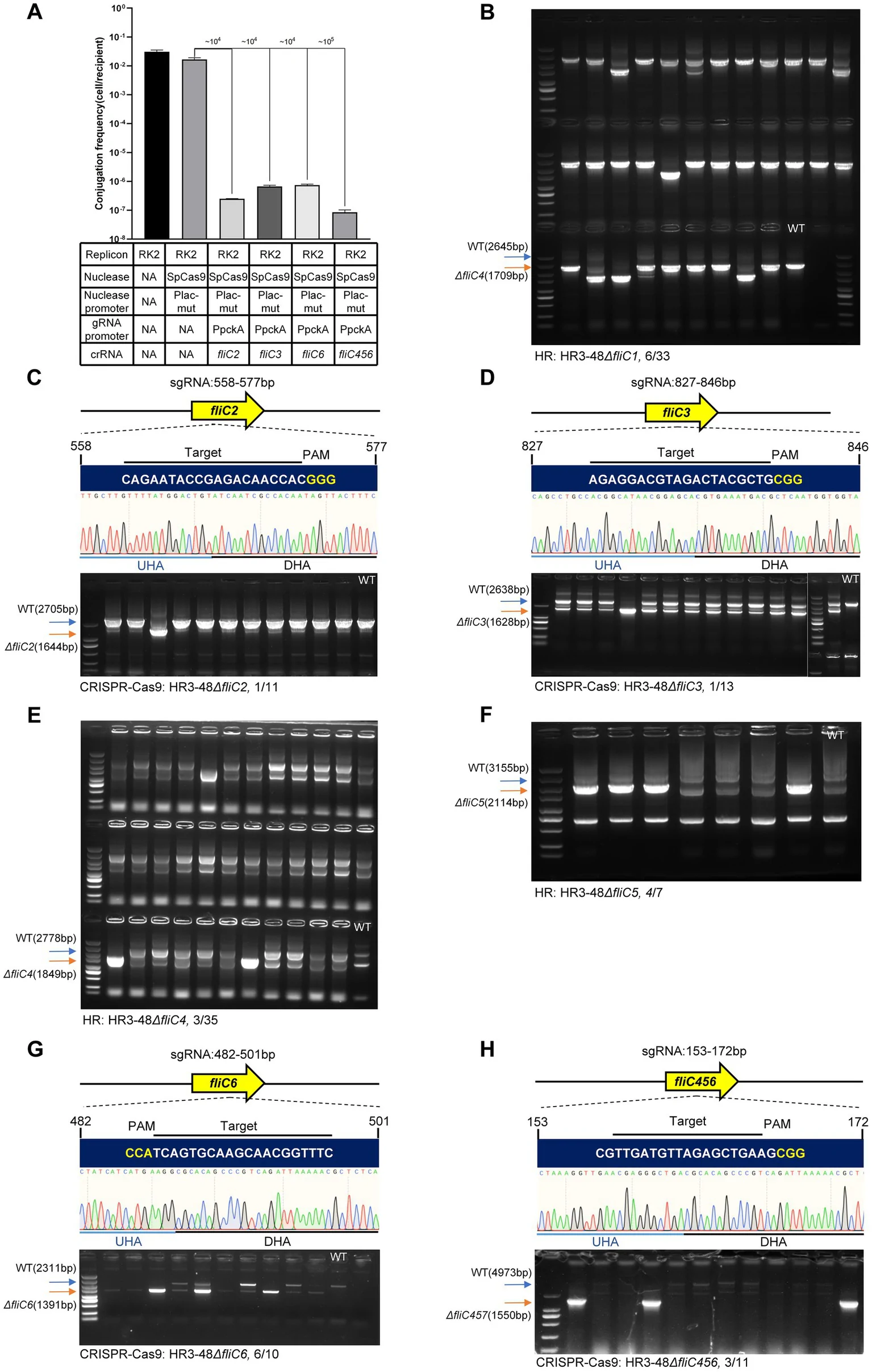
Using a homologous recombination-assisted CRISPR-Cas9 system and pK18mobsacB-mediated seamless genome editing to knockout *fliC* genes in *Pantoea stewartii* subsp. *indologenes* HR3-48. **(A)** Transformation efficiency resulting from conjugation of plasmids without or containing the SpCas9 nuclease, and with or without the combined expression of crRNA targeting *fliC*s. NA, not applicable; **(B,E,F)** pK18mobsacB-mediated homologous recombination (HR) to delete *fliC1*, *fliC4,* and *fliC5*, respectively. **(C,D,G,H)** CRISPR-Cas9 editing system-mediated gene knockout (KO) of HR3-48 *fliC*s. Top: nucleotide sequences of *fliC2* targeted by sgRNA; middle: DNA sequencing chromatograms of upstream/downstream homologous arms (U/DHA) of corresponding *fliC*s are provided for KO lines. Bottom: the electrophoretogram of Δ*fliC* mutants verification.

### Flagellins in the conserved *flag-1* were redundantly required for flagella biosynthesis and motility instead of T3SS

Rotary flagella propel bacteria toward more favorable colonization environments (Nedeljković et al., 2021). To determine which *fliC* copy supports HR3-48 motility, we examined swimming, swarming, and filament formation using a library of 15 *fliC*-deletion mutants (Supplementary Table S2) of HR3-48. Deficiency of the entire tandem *fliC456* cassette in the conserved *flag-1* flagellar operon markedly reduced swimming and swarming halos (Figures 3A,B) and abolished detectable flagellar filaments (Figure 3C), while others remained the same phenotype as wild-type strains. In contrast, mutants retaining any one member of the *fliC456* cassette remained flagellated and motile, even lacking all *fliC* genes outside this cassette (Figures 3A–C). These results indicate that the *fliC456* cassette plays a major role in *Pnsi* HR3-48 motility with functional redundancy among FliC4, FliC5, and FliC6. The T3SS structural mutant HR3-48Δ*hrcQ–U* exhibited no significant difference in swimming or swarming compared with the WT strain (Figures 3A,B), indicating that T3SS integrity does not measurably contribute to flagellar motility in this assay.

**Figure 3:**
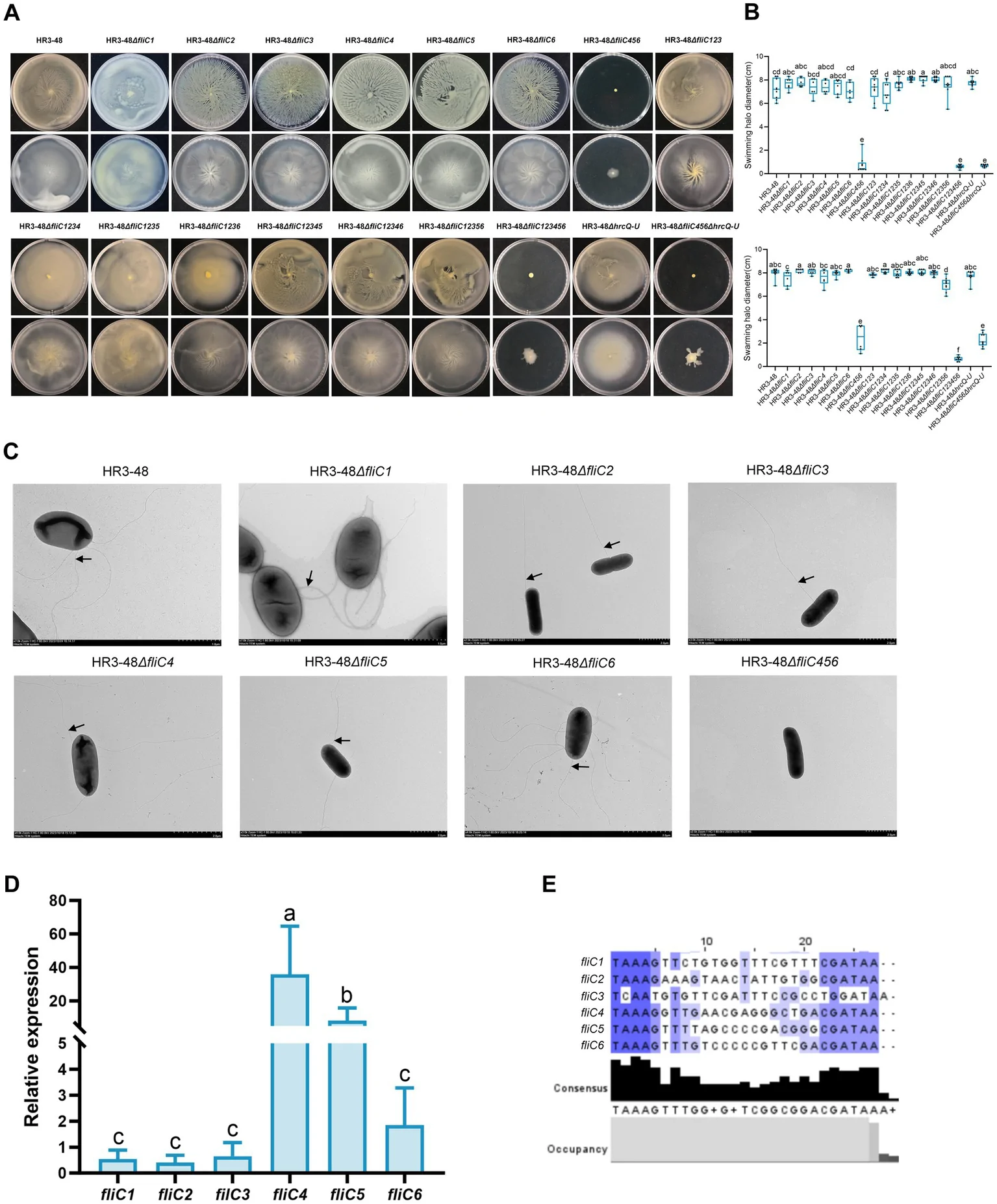
Flagellins in conserved flagellar loci redundantly determine bacterial motility and filament assembly. **(A)** Top: upper channel indicates swimming on 0.2% agar-containing LB medium for 24 h; lower channel indicates swarming on 0.3% agar-containing LB medium for 48 h. Bottom: swimming and swarming of the remainder of *fliC* mutants and HR3-48Δ*hrcQ–U*. **(B)** Swimming and swarming halo diameters measured by ImageJ. Box plots show the median (center line), interquartile range (box), and whiskers (1.5 × IQR); dots indicate individual biological replicates (*n* = 6 or 9). Different letters indicate statistically significant differences (one-way ANOVA followed by Tukey’s multiple comparisons test, *p* < 0.05); **(C)** electron microscopy images of HR3-48 and relevant *fliC* mutants; the black arrows point to the flagellar filament. WT cells showed rod-shaped peritrichous flagella; only triple mutants of *fliC456* exhibited abolishment of the flagellum. Bar, 2 μm. **(D)** Expression profiles of HR3-48 *fliC* genes, data were measured via qRT-PCR and analyzed by the 2^-ΔCt^ method against *gyrB*. Relative expression was determined by comparing each *fliC* gene with the *gyrB* gene expression. Data are means of three biological replicates; error bars represent SD. Different letters denote significance based on one-way ANOVA with Tukey’s test (*p* < 0.05). **(E)** Multiple alignment and promoter motif prediction of the 300-bp upstream regions of individual HR3-48 *fliC* genes using Jalview. Putative σ^28^- and σ^70^-associated promoter motifs are indicated. Consensus-matching nucleotides are shown in blue and the consensus histogram below the alignment represents the percentage of the most frequent nucleotide at each aligned position.

We next examined whether this functional hierarchy was associated with differential *fliC* expression and promoter organization. The *fliC456* cassette had substantially higher transcript levels than the other *fliC* paralogs, consistent with its dominant role in flagellum assembly and motility (Figure 3D). Promoter prediction within the 300-bp upstream regions of individual *fliC* genes identified distinct σ-factor-associated motifs. All *fliC* genes contained putative σ^28^-dependent promoter elements, consistent with their assignment as flagellar structural genes. In addition, the single-copy *fliC1* and *fliC3* loci harbored predicted σ^70^ motifs, suggesting potential condition-dependent regulation (Supplementary Figure S3B). By contrast, the tandem *fliC456* genes were associated primarily with σ^28^-type promoter motifs (Figure 3E; Supplementary Figure S3B). Together, the expression, promoter prediction, and mutant phenotypes support that the conserved *flag-1 fliC456* cassette functions as the dominant motility module in HR3-48, whereas other *fliC* paralogs are less engaged under the tested laboratory conditions.

### Flagellins within *flag-1* required for plant immunity in *Nicotiana benthamiana*

Phytopathogens use T3SS to inject effectors into host cells to suppress MTI, while flagellin acts as both a virulence factor and a dominant MAMP (Deng et al., 2017; Cheng et al., 2021). Mutant targeting the *fliC456* cascade markedly reduced ROS production relative to wild type, whereas other single *fliC* mutants had minimal effects (Figure 4A; Supplementary Figure S4), identifying the *flag-1*-encoded *fliC456* cassette as the principal MTI elicitor in *N. benthamiana* under our assay conditions. In a *Pst* DC3000 challenge-inoculation assay, we initially used 1 × 10^8^ cfu/mL, a concentration commonly used in *N. benthamiana* HR assays (Luo et al., 2023). However, HR3-48 at 1 × 10^8^ cfu/mL pre-infiltration triggered necrosis in *N*. *benthamiana* at 24 h post-inoculation (Figure 4B), we therefore reduced the pretreatment concentration to 5 × 10^7^ cfu/mL, at which HR3-48 and its derivatives failed to elicit HR after 48 h (Figure 4C). Under this condition, pretreatment with WT HR3-48 and the T3SS-proficient *fliC* mutants, HR3-48Δ*fliC456*, did not attenuate DC3000-triggered HR even after 72 h (Figure 4C), indicating that flagellin-derived MTI was not sufficient to suppress the challenge HR in a T3SS-proficient HR3-48 background. In contrast, the T3SS-null mutant HR3-48Δ*hrcQ–U* reduced the HR frequency to 5 × 10^7^ cfu/mL, whereas this suppression of HR was lost in the double mutant Δ*fliC456*Δ*hrcQ–U* (Figures 4C,D), indicating that *fliC456*-dependent MTI is required for HR attenuation in the absence of a functional T3SS. These data suggest that FliCs within the *flag-1* and the PSI-1 T3SS of HR3-48 jointly shape immune outcomes in *N. benthamiana*, possibly through T3SS-delivered effectors that suppress host immunity. Whether the same relationship operates in cereal hosts remains to be tested.

**Figure 4:**
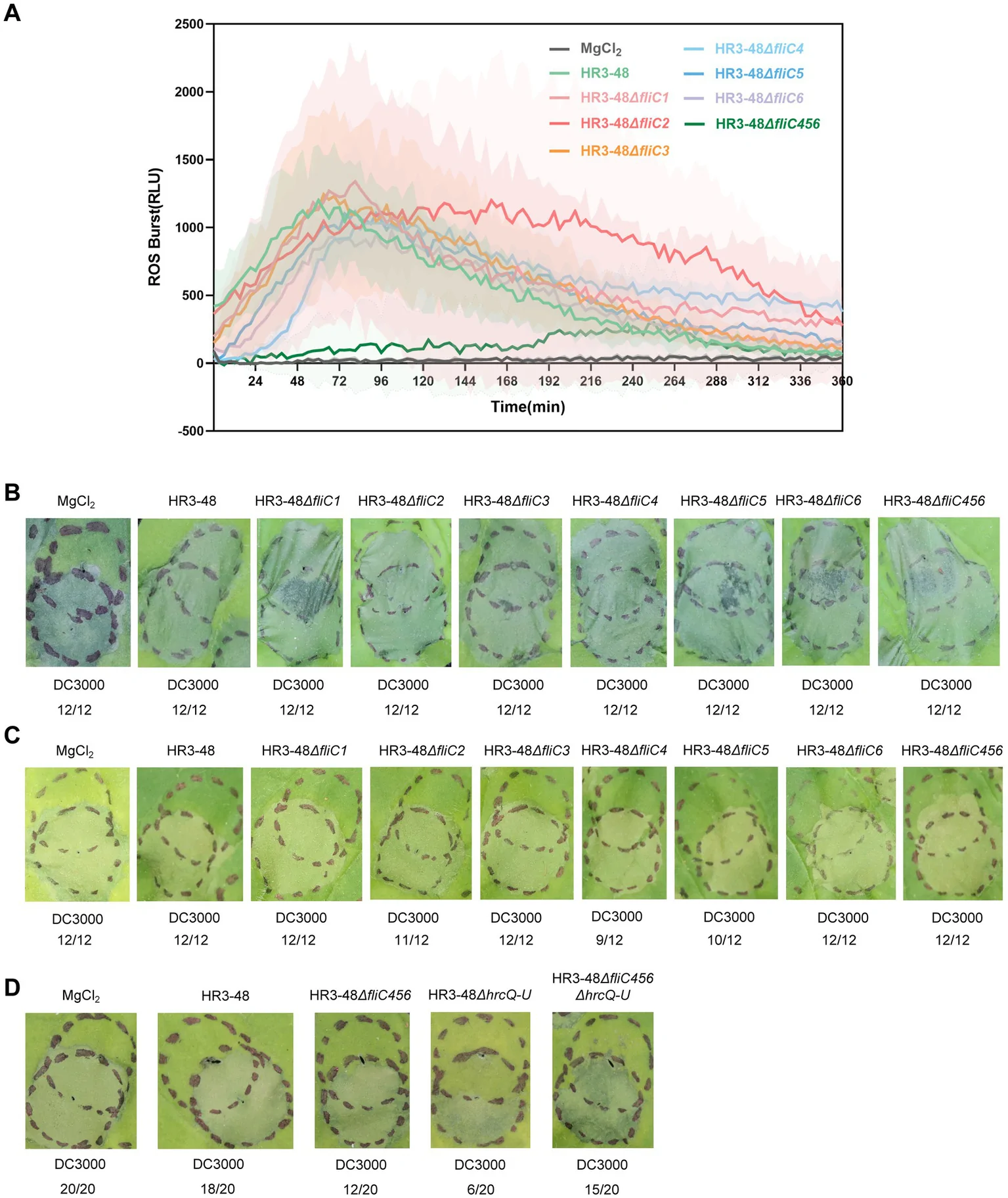
Flagellins located in *flag-1* were required for plant immunity. **(A)** ROS burst assay of strain HR3-48 and *fliC*-related mutants. Bacteria were infiltrated at 1 × 10^8^ cfu/mL into *N. benthamiana* leaves and assayed for ROS burst using L-012 chemiluminescence 12 h later. 10 mM MgCl_2_ was defined as the negative control. RLU indicates relative light units. Lines indicate the mean ROS signal; the shaded area with the corresponding color for each treatment represents the mean ± SD from three biological replicates (each replicate averaged from 12 leaf discs of four plants). **(B)** Challenge inoculum HR assays for functional MTI initially infiltrated *N. benthamiana* leaves at 1 × 10^8^ cfu/mL of the HR3-48 and derivatives (upper circles). The HR-inducing strain *Pst* DC3000 was challenge-inoculated after 6 h at 1 × 10^7^ cfu/mL (lower circles), partially overlapping within the area pre-treated with the *P. stewartii* strains. Presence or absence of confluent cell death in the overlapping region was evaluated and photographed 72 h after *Pst* DC3000 infiltration. Fraction under each image indicates the number of times that HR was present relative to the total number of tested inoculations. **(C)** Challenge inoculum HR assays of HR3-48 and derivatives: bacterial suspensions were infiltrated into *N. benthamiana* leaves at 5 × 10^7^ cfu/mL as described in **(B)**. Data are means of three biological repeats in **(B)** and **(C)**. **(D)** Comparison of HR3-48Δ*hrcQ–U* and HR3-48Δ*fliC456* for ability of MTI elicitation to inhibit challenge inoculum HR. Bacteria were infiltrated at 1 × 10^7^ cfu/mL into *N. benthamiana* leaves; representative leaves with or without disease lesions were photographed 72 h later. Buffer treatment was 10 mM MgCl_2_. Experiments were performed with three biological replicates.

### Pathogenicity of *Pantoea stewartii* predominantly requires T3SS-dependent virulence

HR3-48, isolated from rice cv. Dular seed, did not influence seed germination rate (Figure 5A). Given that *P. stewartii* subsp. *indologenes* has been identified as a pathogen from maize and rice leaf blight, we assessed its pathogenicity *in planta*. We detected the infection assay using leaf-cutting inoculation (Tang et al., 2022). On the non-resistant rice cultivar JG30, the HR3-48 disease lesion length averaged 3.9 cm at 14 dpi, compared with 1.9 cm and 1.5 cm for the T3SS-null mutants HR3-48Δ*hrcQ*–*U* and HR3-48Δ*fliC456*Δ*hrcQ*–*U*, respectively, and was significantly shorter than that of the wild-type strain (Figures 5B,C). Therefore, the Hrc-Hrp T3SS in *Pnsi* HR3-48 is required to achieve full virulence in rice. Deletion of *fliC3* or only the tandem *fliC456* cassette did not reduce rice lesion length to the extent of Δ*hrcQ–U*; thus, flagella are likely dispensable in this context.

**Figure 5:**
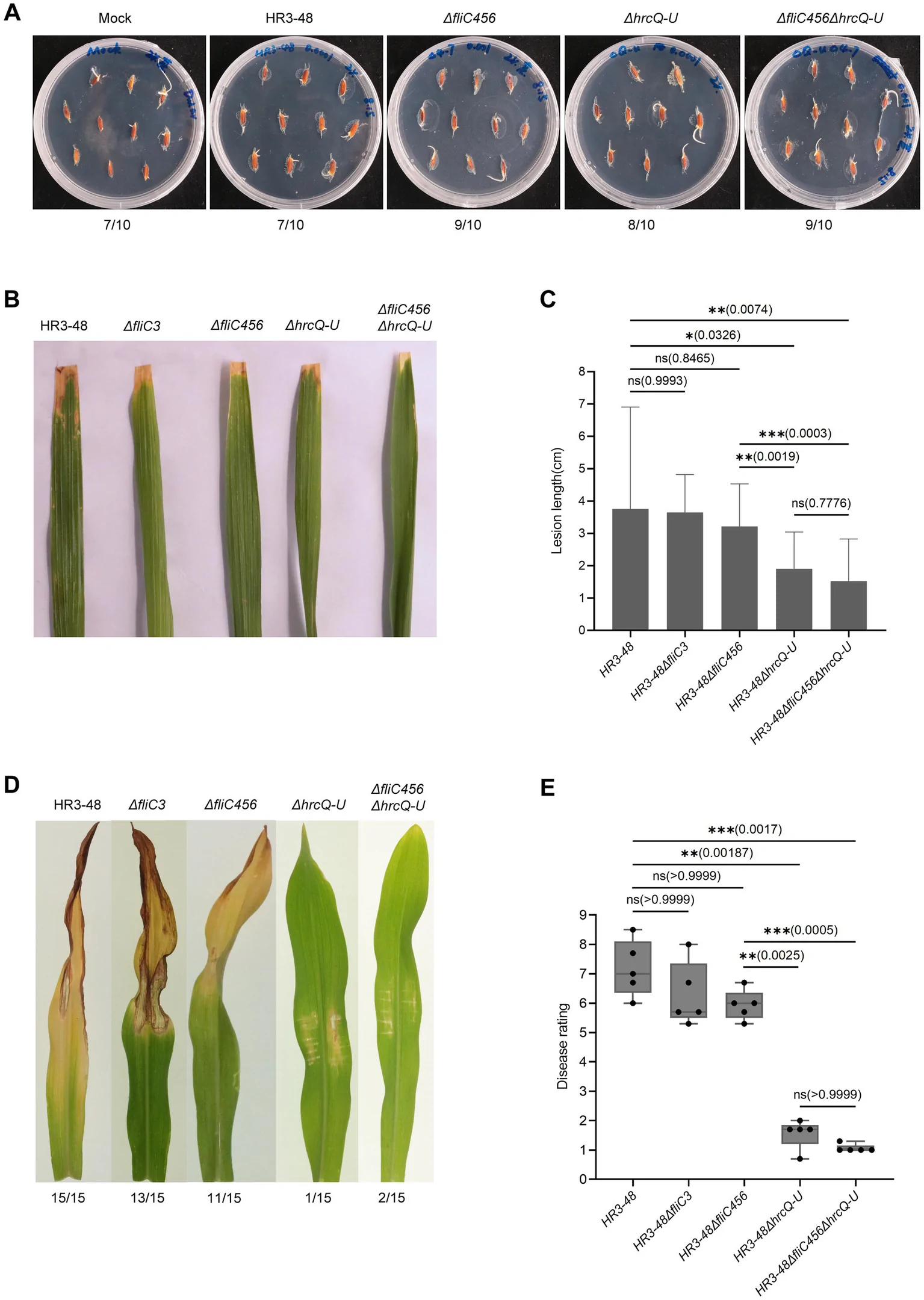
Rice and maize pathogenicity assays. **(A)** Bacteria did not affect the germination rate of Dular rice seeds at 1 × 10^6^ cfu/mL and 1 × 10^5^ cfu/mL after placing 10 treated seeds on 1/2 MS medium supplemented with 0.5% sucrose. Each treatment was performed in triplicate, with the experiment repeated twice. **(B,C)** Rice foliage (4-week-old) was infected by infiltrating suspensions (1 × 10^9^ cfu/mL) of *Pnsi* HR3-48 and its derivatives. Streaking and necrosis were observed at 14 dpi. One-way ANOVA followed by Tukey’s multiple comparisons test (*p* < 0.05) was performed using GraphPad PRISM 8.0.2. Significance levels between groups: ns, not significant; *, *p* < 0.05; **, 0.0005 < *p* < 0.01; ***, 0.0001 < *p* < 0.0005; ****, *p* < 0.0001. **(D,E)** Approximately 5 × 10^8^ cfu/mL of HR3-48 and related mutants were inoculated into maize leaves (8-day-old) and assayed at 14 dpi. Symptoms were rated from 1 to 9 as described by Michener et al. (2003). Wild-type strain showed obvious virulence. Meanwhile, the Δ*hrcQ–U* mutant strain showed diminished disease symptoms (disease rating of 1.5). Lesion lengths and disease ratings were quantified from independent biological replicates (rice: three leaves per plant × three plants, 9 replications; maize: three leaves per plant × five plants, 15 replications). Maize disease ratings are shown as individual biological replicates with median and interquartile range. Statistical significance was assessed by GraphPad PRISM 8.0.2 using the Kruskal–Wallis test followed by Dunn’s multiple-comparisons test (*p* < 0.05); adjusted *p*-values are indicated in the figure.

Eight-day-old hybrid Jubilee maize seedlings were manually inoculated and evaluated for virulence after 2 weeks, following the previous method with some modifications (Michener et al., 2003). The wild-type strain was virulent, with an average disease rating of 6.5. A disease rating of 9 represents dead or near-dead plants (Mubeen et al., 2017). The disease rating for the Δ*hrcQ–U* mutant was 1.5, with limited symptoms and spread (Figure 5D). HR3-48Δ*fliC456* had a rating of 5, with reduced but abundant spread and symptoms similar to WT. Meanwhile, HR3-48Δ*fliC456*Δ*hrcQ–U* had little lesion expansion that was assigned a disease rating of 1. HR3-48Δ*fliC3* inoculation resulted in indistinct phenotypes compared to the wild-type strain (Figures 5D,E). These data indicate that *P. stewartii* subsp. *indologenes* HR3-48 is pathogenic on rice and maize, with the T3SS apparatus dominating to facilitate virulence rather than flagellins.

### Residue 19^th^ of flg22 is the key amino acid for specific plant immune system recognition

We investigated whether the polymorphic flagellins, which result in diverse substitutions of flg22 residues, functionally initiate divergent immune signaling pathways. flg22^PanM^ in FliC3 and flg22^PanE^ in other FliCs were extracted from six FliC proteins of *Pnsi* HR3-48, which had polymorphic substitutions comapred with *P. aeruginosa* PAO1 flg22^Pae^ mainly at the 1^st^, 5^th^, 7^th^, 19^th^, and 20^th^ residues, but only altered at the 19th residue between flg22^PanM^ and flg22^PanE^ (Figure 6A). flg22^PanM^ inhibited *P. syringae* pv. *tomato* DC3000-induced HR, whereas flg22^PanE^ abolished this ability (Figure 6B). flg22^PanM^ induced ROS burst in *A. thaliana* (Figure 6C; Supplementary Figure S5A), *N. benthamiana* (Figure 6D; Supplementary Figure S5B) and Dular rice leaves (Figure 6E; Supplementary Figure S5C), while ROS production induced by flg22^PanE^ was dramatically reduced compared to that induced by flg22^Pae^, even at a higher 100 nM peptide concentration, excluding a dose-dependent effect (Figure 6D; Supplementary Figures S5D,E). However, flg22^PanM^ and flg22^PanE^ suppressed *A. thaliana* seedling growth similarly to the positive control flg22^Pae^ (Figures 6F,G), supported by the ROS burst kinetics in *A. thaliana* (Figure 6C), indicating that flg22^PanE^ is not completely inactive across plant species, probably due to the FLS2 receptor specificity. In summary, the 19th residue of flg22 is critical for host immune responses. Remarkably, the differential ROS and HR but comparable seedling growth inhibition suggest that residue-19 variation primarily alters the strength or early kinetics of flg22 perception rather than fully abolishing FLS2-dependent signaling.

**Figure 6:**
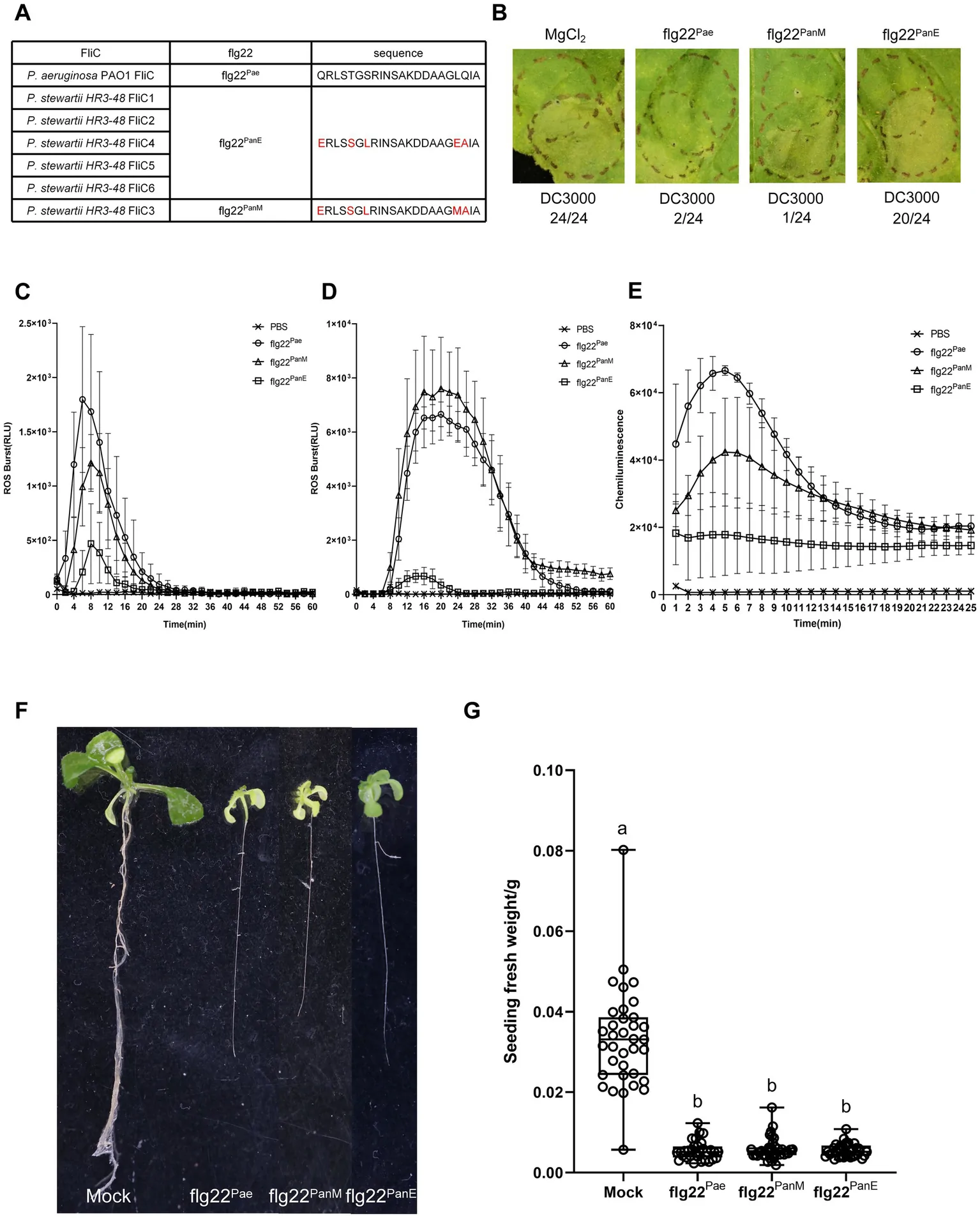
The 19^th^ amino acid of flg22 is the key amino acid for specific plant immune system recognition. **(A)** Amino acid sequence alignment of flg22 variants from HR3-48, with comparison of flg22^Pae^ from *P. aeruginosa* PAO1. Polymorphic substitutions were marked in red, mainly at the 1^st^, 5^th^, 7^th^, 19^th^, and 20^th^ residues among the three peptides, while only Leu^19^ varied between flg22 variants from HR3-48. **(B)** Challenge-inoculation HR assays for functional PTI were conducted by infiltrating *N. benthamiana* leaves with 100 nM flg22 peptides (upper circles). After 6 h, an overlapping inoculation of 1 × 10^7^ cfu/mL of the *Pst* DC3000 (lower circles) was made. The fraction under each image indicates the number of times that the HR was present in relation to the total number of inoculations. **(C)** ROS production induced by flg22^PanE^ and flg22^PanM^ at 100 nM in *A. thaliana*. Analysis represents the mean value (n = 12 leaf discs of 4 plants per treatment) recorded every 2 min from three biological replicates. Statistical significance was determined using a one-way ANOVA followed by a Tukey’s test (*p* < 0.05). **(D)** ROS production induced by flg22^PanE^ and flg22^PanM^ at 100 nM in *N. benthamiana*. At least 12 leaf discs from three independent plants were used per treatment, and the experiment was independently repeated three times. Statistical significance was analyzed using a one-way ANOVA followed by Tukey’s test (*p* < 0.05). **(E)** ROS burst measurement in rice cultivar Dular. Three rice leaf discs were immersed in 100 nM flg22 peptides, and ROS generation was assayed for 25 min. **(F,G)** SGI assays for the *Arabidopsis* seedlings Col-0 upon treatments with 100 nM positive control flg22^Pae^ and flg22^PanE^, flg22^PanM^ variants. Fresh seedling weights (mean ± SE) are for 12 seedlings per treatment, measured after 10 d of growth in 1/2 MS medium supplemented with PBS (mock). Different letters indicate significant differences in seedling weights of Col-0 (*p* < 0.05, one-way ANOVA, Tukey’s test). All experiments were repeated three times with similar results.

## Discussion

The bacterial flagellum and the virulence-associated injectisome that bacteria use for locomotion or for the translocation of virulence factors into eukaryotic host cells are structurally related nanomachines widespread among many Gram-negative bacterial pathogens and symbionts (Deng et al., 2017). T3SS mediate structure assembly and extracellular protein transfer (Erhardt et al., 2010); non-flagellar T3SS (NF-T3SS) translocate effector proteins. The intricate interplay between NF-T3SS, simplified as T3SS, and flagellin-based MAMPs is extensively studied in plant-pathogen interactions (Soscia et al., 2007; Wei et al., 2013; Worrall et al., 2023; López-Pagán et al., 2025). Exploring *P. stewartii* and *P. agglomerans* provides insights into T3SS and T3Es function (Gentzel et al., 2022; Ham et al., 2006; Moretti et al., 2021; Nissan et al., 2019). However, the coordination of T3SS and another key virulence factor, flagellin, in *P. stewartii* remains unclear. In diverse bacteria, type III secretion and flagellar systems are oppositely regulated in a condition- and stage-dependent manner. For example, in the bacterium *Erwinia amylovora*, the T3SS-positive sigma factor HrpL downregulates flagellar biogenesis under *hrp*-inducing conditions, illustrating inverse regulation between T3SS deployment and motility-associated functions (Cesbron et al., 2006). Transcriptome analyses in *Xanthomonas citri* showed that the master regulator HrpG acts as a negative regulator of motility while activating the *hrp* program, consistent with condition-dependent allocation of resources between motility and host interaction (Guo et al., 2011). However, reciprocal regulation is also possible: loss of the flagellar filament (Δ*fliC*) in *P. aeruginosa* increases T3SS transcription and ExoS secretion, whereas a non-motile but flagellated Δ*motAB* mutant does not, implicating that flagellar assembly rather than motility could antagonize the T3SS and that a negative crosstalk exists between these two systems. In addition, the GacA response regulator negatively affects the T3SS, while it was previously reported as a positive regulator for motility (Goodier and Ahmer, 2001; Soscia et al., 2007). Yuan et al. (2015) demonstrated in *D. dadantii* that the flagellar master regulator FlhDC activates HrpL-dependent T3SS expression through EcpC–RpoN, FliA–YcgR, and RsmB–RsmA pathways. Ghasemi et al. (2025) further identified c-di-GMP/CdeR signaling as a shared node controlling T3SS expression and swimming motility in *Dickeya dadantii*. Ward et al. (2025) showed that c-di-GMP binding to the *P. syringae* T3SS ATPase HrcN controls translocation of specific T3Es (Ward et al., 2025). Recent single-cell analyses in *P. syringae* revealed a counter-regulated division of early, host-proximal T3SS^ON^/Flagella^OFF^ cells and later T3SS^OFF^/Flagella^ON^ subpopulations accumulation during apoplastic colonization, supporting that T3SS effectors can function as ‘common goods’ that enable motile, flagellated subpopulations to expand and disseminate despite flagellin immunogenicity (López-Pagán et al., 2025). In HR3-48, Δ*hrcQ–U* retained wild-type motility, but whether *fliC456* disruption reciprocally alters PSI-1 expression or WtsE secretion remains to be tested. In *P. syringae*, the extracytoplasmic function sigma factor σ^22^ ortholog AlgU reduces *fliC* expression in *P. syringae*-plant interactions to dampen FLS2-triggered immunity and facilitate host colonization (Bao et al., 2020). At the epitope level, flagellin polymorphism can modulate FLS2-dependent immunity without measurable motility costs, as shown for flg22 and/or flgII-28 allelic variation in *P. syringae* and within-pathovar flagellin polymorphism in *X. campestris* pv. *campestris* (Sun et al., 2006; Clarke et al., 2013). Pathogens can also evade flagellin perception by FLS2 post-translationally. In *P. syringae*, AprA degrades immunogenic flagellin monomers to reduce FLS2 recognition while largely sparing polymerized flagella (Bardoel et al., 2011; Pel et al., 2014). Together, these precedents support that HR3-48 couples PSI-1 T3SS-dependent virulence with a diversified flagellin repertoire to navigate the motility-immunity trade-off, although the regulatory linkage between these modules remains unresolved. Here, we examined how *P. stewartii* subsp. *indologenes* HR3-48 uses a conserved PSI-1 T3SS and multiple flagellin paralogs to modulate plant immunity and establish infection. Our data support functional partitioning between these systems: PSI-1 is primarily required for pathogenicity and immune modulation, whereas the tandem *fliC456* cassette is required for flagellar filament assembly and motility. In addition, the distinct immune activities of *fliC3*-derived flg22^PanM^ and *fliC456*-associated flg22^PanE^ indicate that residue-19 variation in flg22 can tune flagellin-triggered immune perception.

*Pnsi* HR3-48 has a streamlined T3SS architecture compared to *Pnss* DC283 (Figure 1A), with only the plant-pathogenic PSI-1 system rather than additional insect vector adaptation systems (Correa et al., 2012). This suggests an evolutionary trade-off and potentially reflects its broader host range. Notably, *P. stewartii* has been proposed as a comparatively “minimal” effector system in which an AvrE-family effector (WtsE) can be essential for pathogenicity, and few additional effectors have been shown to contribute measurably to virulence (Roper, 2011). The conserved *wtsE*–*wtsF* effector-chaperone cassette across *Pnsi* strains aligns with the virulence role of WtsE established in *Pnss* (Ham et al., 2006). The diverse effectors in different strains suggest *P. stewartii* relies on a distinct effector repertoire for host-specific adaptation (Figure 1A). At 5 × 10^7^ cfu/mL, the wild-type strain likely delivers sufficient effectors to suppress MTI, whereas the Δ*hrcQ–U* mutant, defective in effector translocation, fails to generate robust HR induction upon *Pst* DC3000 challenge (Figures 4B–D), which may be suppressed by flagellin-activated functional MTI. Such phenotypes mirror inverse regulation between the flagellar and T3SS genes in *P. aeruginosa* (Soscia et al., 2007).

The T3SS structural gene *hrcQ–U* was indispensable for pathogenicity in rice and maize (Figures 5B–E). It is consistent with observations in *P. syringae* and *X. oryzae*, where *hrcQ* mutants lose HR induction in non-hosts and virulence in hosts (Wei et al., 2013; Zhang et al., 2013). However, virulence attenuation was more severe in T3SS mutants than flagellar mutants (Figures 5B,D), indicating that T3SS helps suppress plant immunity. The slight virulence loss in Δ*fliC456* mutants suggests a modest contribution of *fliC456*-dependent flagellar function to disease development under our assay conditions, which differs from the stronger colonization role of flagella-dependent surface motility reported in *P. stewartii* subsp. *stewartii* due to the species-specificity (Herrera et al., 2008). However, complete avirulence of the Δ*fliC456*Δ*hrcQ–U* polymutant indicates that PSI-1 and *fliC456* jointly contribute to pathogenicity in HR3-48 (Figures 5B,D). This interpretation is consistent with previous studies showing that T3SS effectors suppress PTI, including flagellin-triggered immunity (Kvitko et al., 2009), although we did not directly test tissue colonization by HR3-48 flagellar mutants. The retained virulence of Δ*fliC3* mutants suggests that FliC3 plays a primary role in immunomodulation rather than motility.

The *fliC* gene expansion via duplication in HR3-48 indicates evolutionary adaptation through genetic redundancy and differentiation. Tandem *fliC456* genes in the *flag-1* locus showed redundancy for motility but collectively abolished most MTI elicitation (Figures 3A, 4A). This contrasts with endophytic bacteria, where flagellins influence motility but avoid immune activation (Buschart et al., 2012). Their high σ^28^-driven expression ensures filament assembly (Figure 3D; Supplementary Figure S3B), while genetic redundancy protects this from mutational loss (Soutourina and Bertin, 2003; Faulds-Pain et al., 2011). The scattered *fliC* genes (e.g., *fliC3*) with dual σ^28^/σ^70^ regulation and lower expression may have ancillary roles in adhesion or biofilm formation (Jung et al., 2019). This highlights an evolutionary strategy to decouple motility from immune recognition. The bacterium may maintain propulsion while potentially concentrating less-immunogenic epitopes in a redundant, high-expression locus that can evolve under host selection pressure. Polymorphism at the 19th residue in flg22 illustrates host-specific immune recognition. Flg22^PanM^ (L19M) strongly elicits ROS in *A. thaliana*, *N. benthamiana,* and rice and suppresses *Pst* DC3000-induced HR in *N. benthamiana*, while flg22^PanE^ (L19E) showed markedly reduced or little activity in these early immune assays. This is consistent with structural studies highlighting the role of Leu^19^ in stabilizing FLS2-BAK1 complexes (Sun et al., 2013; Wei et al., 2020). However, both peptides inhibited *Arabidopsis* seedling growth. This pattern may reflect differences in FLS2 ligand specificity or response thresholds among plant species and immune readouts (Gómez-Gómez et al., 1999; Belkhadir et al., 2014). Often flanked by mobile elements (Figure 2C), the prevalence of invalid flg22^PanE^ variants potentially indicates horizontal acquisition of novel epitopes for immune evasion. Although the FliC3-derived flg22^PanM^ peptide showed immune activities, deleting *fliC3* did not measurably affect motility or virulence under our tested conditions. Thus, the contribution to functional flagella or host colonization of full-length FliC3 remains unresolved. Together, comparative genomics revealed substantial strain-level variation in both virulence and flagellar loci. T3SS repertoires vary in presence and composition of PSI-1 to PSI-3, whereas the conserved *flag-1* locus differs in gene organization and *fliC* copy number (3–7), accompanied by three flg22 variants (L19E/L19Q/L19M). Whether particular combinations of T3SS repertoire, flagellin architecture, and flg22 identity co-evolve to determine host range, immune visibility, or pathogenic strategy remains an important question for further comparative functional analyses across *P. stewartii* lineages. *Pnsi* HR3-48 uses an integrated virulence strategy where T3SS effector delivery and flagellar functionality are coordinated through evolutionary refinement. In summary, our comparative genomics and mutant analyses support a more limited model in which PSI-1 T3SS integrity is a major determinant of virulence in rice and maize, whereas the tandem *fliC456* cassette primarily governs flagellar filament assembly and motility (Figure 7). Immune assays further show that *fliC456* contributes strongly to ROS induction in *N. benthamiana* and that the flg22 residue-19 polymorphism modulates immune readouts across plant species in *A. thaliana*, *N. benthamiana* and rice. The different activities of flg22^PanE^ in *N. benthamiana*, rice, and Arabidopsis are consistent with previous studies showing that FLS2 homologs such as AtFLS2, SlFLS2, OsFLS2, and VvFLS2 differ in ligand specificity (Mueller et al., 2012; Trdá et al., 2014). Therefore, flg22^PanE^ should be interpreted as a weak or context-dependent agonist rather than a universally immune-evasive epitope. These experimentally supported relationships and the unresolved interplay between the PSI-1 T3SS and flagellin-mediated immunity are summarized in Figure 7. However, the immune hierarchy observed in *N. benthamiana* should not be directly extrapolated to cereal hosts. Future studies should focus on exploiting effector repertoire identification with secretion assays and analyses for effector-specific immune mechanisms in host plants to offer sustainable disease management strategies against *Pantoea* pathogens.

**Figure 7:**
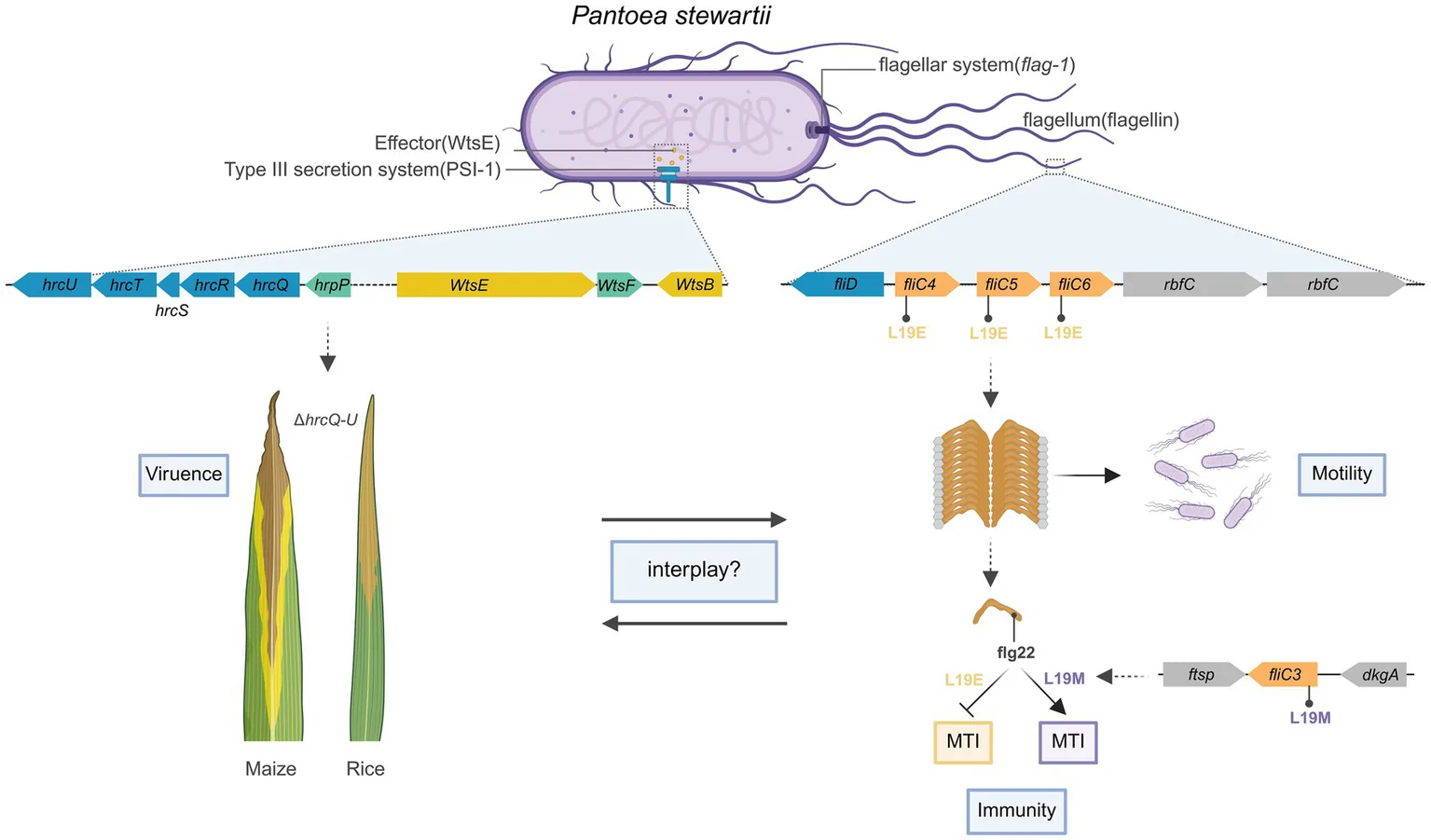
Model of T3SS-mediated virulence and flagellin-triggered immunity in *Pantoea stewartii* subsp. *indologenes* HR3-48. Genome-informed dissection of *Pantoea stewartii* subsp. *indologenes* HR3-48, showing that PSI-1 T3SS integrity (Δ*hrcQ–U*) is required for rice and maize necrosis; the tandem *fliC456* cassette redundantly contributes to flagellar assembly, motility, and represents a major source of flagellin-triggered MTI. Natural variation at residue 19 (L19M and L19E) of flg22 peptides in *Pnsi* HR3-48 further modulates distinct immune readouts. The potential interplay between the T3SS and flagellin-mediated immunity remains to be resolved. Created with BioRender.com.

## Data Availability

The datasets presented in this study can be found in online repositories. The names of the repository/repositories and accession number(s) can be found in the article/Supplementary material.

## Footnotes

1
https://www.ezbiocloud.net/

2
http://ggdc.dsmz.de/distcalc2.php

3
http://crispor.tefor.net/
